# Data shopping in an open marketplace: Introducing the Ontogrator web application for marking up data using ontologies and browsing using facets

**DOI:** 10.4056/sigs.1344279

**Published:** 2011-04-29

**Authors:** Norman Morrison, David Hancock, Lynette Hirschman, Peter Dawyndt, Bert Verslyppe, Nikos Kyrpides, Renzo Kottmann, Pelin Yilmaz, Frank Oliver Glöckner, Jeff Grethe, Tim Booth, Peter Sterk, Goran Nenadic, Dawn Field

**Affiliations:** 1 School of Computer Science, University of Manchester, Manchester, UK; 2 Natural Environment Research Council Environmental Bioinformatics Centre, NERC Centre for Ecology and Hydrology, Wallingford,UK; 3 The MITRE Corporation, Bedford, MA, USA; 4 Department of Applied Mathematics and Computer Science, Ghent University, Ghent, Belgium; 5 DOE Joint Genome Institute, Walnut Creek, CA, USA; 6 Microbial Genomics Group, Max Planck Institute for Marine Microbiology, Bremen, Germany; 7 Jacobs University Bremen GmbH, Bremen, Germany; 8 University of California San Diego, La Jolla, CA, USA; 9 Molecular Evolution and Bioinformatics Group, NERC Centre for Ecology and Hydrology, Wallingford, UK

## Abstract

In the future, we hope to see an open and thriving *data market* in which users can find and select data from a wide range of data providers. In such an open access market, data are *products* that must be packaged accordingly. Increasingly, eCommerce sellers present heterogeneous product lines to buyers using faceted browsing. Using this approach we have developed the Ontogrator platform, which allows for rapid retrieval of data in a way that would be familiar to any online shopper. Using Knowledge Organization Systems (KOS), especially ontologies, Ontogrator uses text mining to mark up data and faceted browsing to help users navigate, query and retrieve data. Ontogrator offers the potential to impact scientific research in two major ways: 1) by significantly improving the retrieval of relevant information; and 2) by significantly reducing the time required to compose standard database queries and assemble information for further research. Here we present a pilot implementation developed in collaboration with the Genomic Standards Consortium (GSC) that includes content from the StrainInfo, GOLD, CAMERA, Silva and Pubmed databases. This implementation demonstrates the power of *ontogration* and highlights that the usefulness of this approach is fully dependent on both the quality of data and the KOS (ontologies) used. Ideally, the use and further expansion of this collaborative system will help to surface issues associated with the underlying quality of annotation and could lead to a systematic means for accessing integrated data resources.

## Introduction

The field of molecular biology is now a data-intensive discipline, which can largely be attributed to recent advancements in ‘omics technologies [1]. Due to the increasing affordability of these technologies, there is now an ever-expanding, increasingly democratized and complex array of distributed data resources for the scientific researcher to contend with. The most recent Nucleic Acids Research (NAR) database special issue states that there are now over 1200 published biological databases in the accompanying online Database Collection [2]. Now, more than ever before, we require better tools to enable researchers to exploit this growing body of information, including the ability to work at the intersection of data held in different resources [3].

### Faceted Browsing

Here we explore an approach – that of faceted browsing – for pulling together and viewing biological data resources in a new way. This approach has been successfully used in eCommerce for managing the exploration of large and complex search spaces. Faceted browsing is the use of information of different types (presented in facets, frames or windows) to quickly compare and select criteria about products [4]. In other words, individual products are placed under multiple classification hierarchies and can therefore be viewed by users in a multitude of ways. This method is particularly prevalent in Web sites that have extensive product catalogues, such as iTunes and Amazon, where items are described by their key attributes like price, manufacturer/publisher or genre.

Consider, for example, visiting Amazon to buy a television. Rather than being presented with a list of thousands of different items, users are asked to narrow down their choice by picking elements from different facets i.e. dimensions that describe the product they are looking for. For televisions, facets might include: screen-size, brand, price-range, refresh-rate and so on. As the user selects particular characteristics, the number of matching candidates in the product catalogue is fed back on the screen and the list becomes more manageable.

The major benefit of faceted browsing, compared to traditional keyword searches, is that users need not have any prior knowledge of either the content or structure of the underlying resources. Research within the information retrieval and human-computer interaction communities has shown faceted browsing to be both popular and effective in data exploration [4].

The field of biology has arguably one of the largest and best product *back catalogues* of any science discipline. In order to utilize these data more effectively, we suggest here that biological data can be packaged-up and described as *data products*. However, unlike a multinational company that can control the descriptions of its product lines, biological data is highly distributed and heterogeneously described. In order to achieve a truly open biological linked-data marketplace, we need standardized and robust descriptions of our data products.

### Annotating data products to support faceted browsing

Many types of biological data still lack informative descriptions – even those that are minimal [5,6] – and while the use of ontologies for annotation is growing, there are still large resources that remain unannotated in any meaningful way. In such cases, it is still possible to extract information directly from a variety of data sources (or literature) to create useful annotations that support navigation and discovery. This can be done by matching content to relevant lists of terms of special interest to users. Consensus lists of such terms from different knowledge domains are collectively known as Knowledge Organization Systems (KOS) and can range from simple glossaries and dictionaries (or controlled vocabularies) through to more complex classification schemes, taxonomies, thesauri, gazetteers and ontologies.

Here we present the Ontogrator web application, where we have used a set of KOS to demonstrate how data can be *marked-up* to create informative facets for search and discovery. We are in particular interested in the use of ontologies as facets. Ontologies can be loosely defined as sets of concepts or terms that also contain explicit relationships between them. Perhaps the best-known example of an applied ontology in the field of Molecular Biology is the Gene Ontology (GO) [7], but there are now a wide range of available ontologies [8] opening up a range of options for future aggregation and *Ontogration* of data.

## Material and methods

The Ontogrator Web application provides a JavaScript GUI (graphical user interface) running within a web browser. This Web application fetches data on demand from a back-end comprising a set of REST (representational state transfer) web services supported by a LAMP (Linux, Apache, MySQL, PHP) software stack. A MySQL database is constructed and indexed specifically to support the functions of the browser GUI.

**Figure 1:**
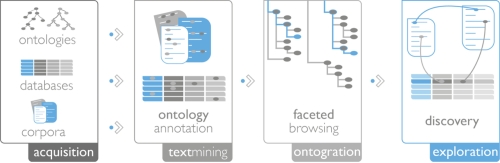
**The Ontogrator platform.** Ontologies, or other KOS, and selected content are processed for use in Ontogrator. After data acquisition and annotation (semantic indexing), browsing services enable exploration and discovery through the web application.

The back-end service (see Figure 1) performs the following key functions: **A - Data acquisition**: ingestion of raw data from primary sources; **B - Semantic indexing**: detecting concepts in the data using text mining; **C - Browsing services**: providing the client with an efficient concept-based retrieval service; **D - Data and facet updates**: periodic refreshing of the underlying resources.

### A - Data acquisition

Data to be imported is converted to tabular format and pre-processed using a PHP script which is customized for each data source. This identifies which columns should be scanned for terms as well as constructing a unique identifier for each record. For example, a data resource with a *habitat* column would be marked for matching against the Environment Ontology. Once the input processors have been constructed, the remainder of the processing is fully automated. The import scripts create appropriate tables in the back-end database to hold both the data and any hits found during semantic indexing.

### B - Semantic indexing

Concept annotation is performed by Terminizer [9], an external Web service that detects mentions of ontological concepts from a given ontology in a given textual passage. Word stemming and phrase rearrangement are employed to spot approximate matches, as well as blacklisting to remove common false positives. In addition to this generic ontology matching service, Ontogrator can call upon other external Web services. For example, in the genomic Ontogrator instance, the uBio [10] service is used for detecting species identifiers and GAZ [11] is used for identification of geo-locations.

### C - Browsing services

A collection of PHP scripts are installed on the server to be called by the Web application. They provide the following functions over a RESTful interface:

- List the available data sources
- List the root concepts of an ontology or taxonomy
- List the *child* concepts of a given ontology concept or taxonomical identifier
- List the concepts which match a given substring (for *autocomplete* when searching)
- Return meta-data about the columns available in a given data source
- List the items from a data source which match any given set of concepts or identifiers
- Give a detailed breakdown of exactly how an item matches against a set of concepts or identifiers.

In our demonstration system, the term lookup is sufficiently fast to provide the user with an interface that is updated in a timely manner. As the speed of the queries depends on straightforward indexing of the underlying database, we anticipate that this would continue to work as more data was added. As all the operations are read-only, server-side replication of the database and/or Web server threads could be employed to maintain performance in the light of increased demand. It may also be useful for people to access these REST services directly, but at this point they are provided solely to feed the web application and we do not advertise them as a public API.

### D - Data and facet updates

As the underlying data sources and ontologies change, these updates need to be reflected in the Ontogrator. For example, scripts in the back-end can be set to update the content of each resource (i.e. add new rows or update entries that have been changed) whenever a new version is published. Only the affected rows need to be re-processed. On the other hand, an update to ontology resources (used for semantic indexing) would necessitate a full re-processing of all data sources as a background batch process.

### Ontogrator Web Interface

**Figure 2:**
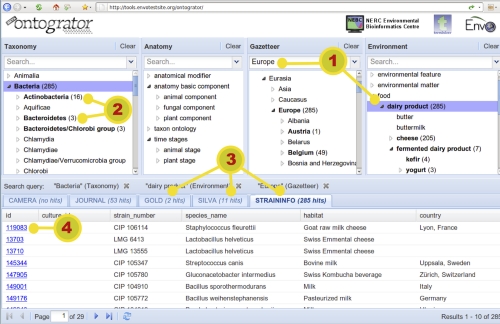
**The Ontogrator Interface.** 1) Users pick facet terms by hierarchical browsing or through keyword search. 2) Continuous feedback about the distribution of hits across the facet helps to guide exploration. 3) The number of hits in all available resources is displayed in real-time. 4) Hits include hyper-links back to the primary source.

The Ontogrator interface, as viewed in a browser, is divided into two principal sections (Figure 2). The top half of the page contains facet browsers, one for each of the available ontologies or taxonomies. The bottom half contains a tabular viewer for displaying results. In between these sections is a toolbar which displays the current search query (i.e. *restrictions* applied in the faceted windows). All sections can be interactively resized by the user.

Individual items in the results table contain a hyper-link back to their entry in the source database. Typically, these entries contain significantly more information than is displayed in the view window of Ontogrator. Additionally, items can be double-clicked to display a pop-up information window which gives details about the exact set of ontological concepts which were associated with this item. This view can be helpful when trying to discover exactly why a particular item has been considered to be a match for the current filters.

The user interface is built using the Ext JS toolkit [12]. This provides for a highly interactive interface composed of familiar widgets such as tree browsers, tables and combo-boxes. The Ext JS library enables the interface to function correctly on all contemporary browsers. The user interface has been engineered to ensure that some results are always visible on screen in order to avoid the common problem of users failing to scroll long pages and thus failing to see the searches in action. The number of visible facets is currently limited to four, as this allows side-by-side display of them without consuming too much page real estate. As discussed previously, the architecture is expected to scale well, so there is no fundamental limit preventing the use of more facets, or, for example, allowing four to be selected from a larger pool.

## Results

### An instance of Ontogrator for the Genomic Standards Consortium (GSC)

To demonstrate the utility of facetted browsing as applied to biological data, we have worked within the Genomic Standards Consortium (GSC) [6,13] to produce an instance of the Ontogrator system populated with content from genomic, metagenomic, marker gene sequences and culture collection databases [14]. The data resources chosen included: 1) the Genomes OnLine Database [15] (GOLD), 2) the Community Cyberinfrastructure for Advanced Microbial Ecology Research and Analysis Database [16] (CAMERA), 3) the SILVA comprehensive ribosomal RNA database [17] and 4) the StrainInfo.net database [18]. Furthermore, we also including text from a selection of 5) PubMed abstracts to illustrate the value of integrating information about molecules, organisms and the literature at the same time. In this instance of Ontogrator, facets were chosen that organize the meta-data content by taxonomy, anatomy, environment and location, the latter two axes of data organization being of special interest to the GSC [3]. We have created these four facets using the following KOS: 1) a species taxonomy (represented by the NCBI taxonomy [19]), 2) anatomy (modeled by MIAA [20]), environmental factors (represented by EnvO [21]) and geographical locations (represented by GAZ [11]).

### Using Ontogrator to explore marked up data

In the screenshot of the Ontogrator online interface as shown in Figure 2, the initial view of the *ontograted resources* shows a default data source and the root terms of the different ontologies. Each time the user adjusts the query by picking one or more terms in a facet, the results table is updated showing items and hit counts from the selected data resource. It is this continual display of visual feedback about the distribution of results that gives users the navigational awareness that enables the successful exploration of unfamiliar data.

In Figure 2, the query retrieves data products from the five data sources that are related to Scandinavian Peninsula and Dairy product. Once a resource is selected as active (e.g., StrainInfo in Figure 2), its instances appear in a conventional tabular form with each row being a direct hyper-link back to the primary resource. The number of hits in each of the non-active resources is also shown, allowing the user to quickly ascertain which of them might also be worth browsing (e.g., there are fourteen hits relating to scholarly publications for the same query in the PubMed database that may be of interest). Additionally, the distribution of hits across the facet is also given, so that the user is guided in their exploration. For instance, if *dairy product* is showing 4 hits, expanding that node might reveal that *cheese* accounts for 1, *milk* for 2 and *fermented dairy product* for 1 of those results. Drilling down through the facet in this way allows the user to adjust the granularity of the query to a level with which they are comfortable.

## Discussion

Here we discuss the main strengths and challenges of the proposed approach, as well as future developments that could be integrated into Ontogrator.

### Searching using the power of ontologies

A major benefit of ontology-driven searching is the automatic broadening of retrieval results, e.g., *look for bone but find tibia*. Such searching is far more powerful than traditional keyword searching in which query terms must match the results explicitly. Using the current content of the Ontogrator genomic instance, a search for *bacterial cultures* in StrainInfo isolated from *dairy products* in *Scandinavian Peninsula* (see Figure 2) returns, among others, an entry from *a culture of ****Leuconostoc**** isolated from ****kefir**** in ****Stockholm***. Even though this result contained none of the chosen query terms, the underlying subsumption hierarchy – of the species taxonomy, the Environment ontology and a geographical location Gazetteer – enabled the system to infer that ***Leuconostoc**** was a kind of ****bacteria***, ***kefir**** was a kind of (****fermented) dairy product*** and ***Stockholm**** was located in the ****Scandinavian Peninsula***. This example clearly shows the potential of semantically-enhanced facets for broadening the search space over conventional, keyword-based information retrieval systems.

Ontogrator facilitates the exploration and discovery of unexpected patterns in concept co-occurrences across facets, which might lead to the generation of novel research hypotheses. It supports both data drill-down to focus the results, and roll-up to generalize the queries. The top levels of the faceted trees give a useful overview of the query distribution over the data set; e.g., which species or countries are overrepresented, and how many hits are available. For example, using the facets provided here, a user could select *habitat=soil* from the Environment dimension and then see how soil samples are distributed across countries by referring to the occurrence of entries now shown in the location facet.

No prior knowledge of either the content or structure of these resources is needed by the users, as the faceted browsing interface provides both the query vocabulary and navigational feedback. In contrast to existing interfaces based on keyword searching, by using ontologies Ontogrator overcomes the *guess the keyword* problem and provides the user with a new yet familiar way to explore distributed data sets in a unified environment.

### Future features

An obvious future direction for the Ontogrator platform to take without any further modification is to increase the number of data resources *ontograted*, either by increasing the number of resources in the GSC portal, or by building other community portals (e.g., model organisms, clinical trials, or environmental data).

In addition to facets based on existing ontologies and taxonomies (which are represented as trees), new facet types could be imagined that use other ways (i.e. KOS) in which to organize data. For example, some facets might be better represented graphically, for example a schematic representation of the human digestive tract for exploring human microbiome project data; or a geopolitical map facet for exploring samples marked up with geolocations. Furthermore, a phylogenetic tree facet can be used to display entries according to their evolutionary relatedness, or a semantic network of concepts can be used to represent dimensions that have not yet been formally represented.

We can also envisage more relaxed matching of resource entries in cases when there are few hits using the standard ontological matching or when different resources have been semantically indexed by different, yet related ontologies. Matches in these cases could be based on semantic distances between pre-computed database annotations and/or user queries. We could use the semantic layer (i.e. ontological annotations) to enable cross-database retrievals through the automated discovery of mappings based on semantic distances between conceptual tags. This approach should provide retrieval of data instances that are, for example, *similar to dairy products* even though the dataset has not been indexed by such tags. For example, a future interface could support functions like *users who searched for this have also searched for this and this*.

### Capturing the user experience

As a third party data aggregator, the quality and accuracy of data annotation is of paramount importance when retrieving data via Ontogrator. The ultimate test for the impact of such systems is the end users. In order to continually improve the application and the data that underpins it, we aim to provide a feedback mechanism where users of the Ontogrator application can rate the appropriateness of data sets based on their search criteria and alert data suppliers of problems with their data.

### Driving ontology development

The Ontogrator could be used to help mature and improve the ontologies it relies upon. More precisely, it could implement a mechanism to provide feedback on terms that have either been overrepresented in data (and may need further specialization) or do not exist in the current hierarchy (e.g., a *term clearing house* can be provided for the submission of new terms to existing ontologies). Similarly, Ontogrator could be open to user-driven updates of annotations/mappings of the Ontograted resources (e.g., a user can indicate that a returned entry is not relevant to a particular query, so the software could have the ability to *learn* e.g., by removing the annotations and/or by re-training the mapping tools.

## Conclusions

We argue that the combined approach of faceted browsing and resource aggregation is an effective solution for aligning and mining information across a collection of related databases. Furthermore, by combining the power of searching over information resources with ontologies, complex distributed data sets can be searched over whilst leveraging the combined knowledge of expert communities.
